# Are tumor-associated micro-angiogenesis and lymphangiogenesis considered as the novel prognostic factors for patients with Xp11.2 translocation renal cell carcinoma?

**DOI:** 10.1186/s12885-020-07696-2

**Published:** 2020-12-02

**Authors:** Wenliang Ma, Jun Yang, Ning Liu, Xiaohong Pu, Feng Qu, Linfeng Xu, Xiaozhi Zhao, Xiaogong Li, Gutian Zhang, Hongqian Guo, Dongmei Li, Weidong Gan

**Affiliations:** 1 Department of Urology, Nanjing Drum Tower Hospital, The Affiliated Hospital of Nanjing University Medical School, No. 321 Zhongshan Road, Nanjing, Jiangsu Province People’s Republic of China 210008; 2 Department of Pathology, Nanjing Drum Tower Hospital, The Affiliated Hospital of Nanjing University Medical School, Nanjing, Jiangsu China; 3 Immunology and Reproduction Biology Laboratory & State Key Laboratory of Analytical Chemistry for Life Science, Medical School, Nanjing University, Nanjing, Jiangsu China; 4 Jiangsu Key Laboratory of Molecular Medicine, Nanjing University, Nanjing, Jiangsu China

**Keywords:** Micro-angiogenesis, Lymphangiogenesis, Renal cell carcinoma, Xp11.2 translocation, TFE3, Prognosis

## Abstract

**Background:**

Tumor micro-angiogenesis and lymphangiogenesis are effective prognostic predictors in many solid malignancies. However, its role on Xp11.2 translocation RCC has not been fully elucidated. Herein, we purposed to explore the correlation between quantitative parameters of tumor-related micro-angiogenesis or lymphangiogenesis and the prognosis of Xp11.2 translocation renal cell carcinoma (Xp11.2 translocation RCC).

**Methods:**

Tissue samples were obtained from 34 Xp11.2 translocation RCC and 77 clear cell renal cell carcinoma (ccRCC) between January 2007 and December 2018. Micro-angiogenesis was detected using CD34 antibody and quantified with microvessel density (MVD) and microvessel area (MVA), while the lymphangiogenesis in RCC was immunostained with D2–40 antibody and assessed using lymphatic vessel density (LVD) and lymphatic vessel area (LVA). The Kaplan-Meier method of survival analysis was used to estimate prognosis, and both univariate and multivariate analysis was performing using the Cox proportional hazards.

**Results:**

The MVD and MVA of Xp11.2 translocation RCC in two detected areas (intratumoral and peritumoral area) were not significantly different from that of ccRCC (all *P* > 0.05). Notably, D2–40-positive lymphatic vessels of Xp11.2 translocation RCC were highly detected in the peritumoral area compared to the intratumoral area. Interestingly, the peritumoral LVD and LVA of Xp11.2 translocation RCC were higher than that of ccRCC (all *P* < 0.05). Furthermore, both intratumoral MVD or MVA and peritumoral LVD or LVA were significantly associated with pT stage, pN stage, cM stage, AJCC stage, and WHO/ISUP grade (all *P* < 0.05). Univariate analysis of Cancer-specific survival (CSS) revealed that CSS was substantially longer in patients with low intratumoral MVD or MVA than in patients with high intratumoral MVD or MVA (*P* = 0.005 and *P* = 0.001, respectively). Lastly, the Cox proportional hazards model in CSS demonstrated that both intratumoral MVD or MVA and peritumoral LVD or LVA were not independent prognostic parameters (all *P* > 0.05).

**Conclusions:**

This study outlines that Xp11.2 translocation RCC is a highly vascularized solid RCC, characterized by rich lymph vessels in the peritumoral area. Quantitative parameters of micro-angiogenesis and lymphangiogenesis could not be considered as novel prognostic factors for patients with xp11.2 translocation RCC.

## Background

In primary tumors, distant metastasis is the most important predictive factor of prognosis in many malignant tumors. The common route of tumor metastasis involves tumor cell invasion and penetration lymphatics and/or blood vessels, subsequently disseminating to distant organs. Furthermore, angiogenesis within the tumor is crucial for tumor growth and metastasis, whereas lymphangiogenesis is a critical factor for tumor progression [1–3]. Growth of the primary tumor mass requires angiogenesis, and new microvessels within tumors are predominant sites for tumor cell entry into circulation due to lack of intact basement membrane and a tight junction between endothelial cells [4]. Vascular endothelial growth factors family members stimulate lymphangiogenesis in tumors thereby enhancing metastatic processes [2, 5].

Xp11.2 translocation renal cell carcinoma (Xp11.2 translocation RCC) is a rare subtype of renal cell carcinoma (RCC), characterized by several different chromosomal translocations involving Xp11.2 and the formation of TFE3 fusion genes, followed by overexpression of TFE3 protein [6]. Patients with Xp11.2 translocation RCC have often presented with advanced stages and demonstrated an invasive clinical course and poor prognosis [7]. In addition, Xp11.2 translocation RCC patients exhibited moderate prolonged enhancement under dynamic contrast-enhanced computed tomography and were more susceptible to lymph node metastasis than those with common subtypes of RCC [8, 9]. Recent studies based on microvessel density (MVD), microvessel area (MVA), lymph vessel density (LVD), and lymph vessel density (LVA) have revealed that micro-angiogenesis and lymphangiogenesis are significant prognostic predictors of RCC or clear cell renal cell carcinoma (ccRCC) [10–13]. However, the role of micro-angiogenesis and lymphangiogenesis within Xp11.2 translocation RCC has not been fully elucidated. Microvessel density (MVD) or LVD, parameter designed to quantify the extent of tumor vascularization or lymphatics, refers to the number of small blood or lymph vessels per tumor area, while MVA or LVA is described as the total lumen area of small or lymph vessels. Nevertheless, due to limited direct studies on Xp11.2 translocation RCC, we quantified micro-angiogenesis and lymphangiogenesis within Xp11.2 translocation RCC using the parameters including MVD, LVD, MVA, and LVA.

Herein, we purposed to explore the correlation between tumor-related microvascular or lymphatic quantitative parameters and the prognosis of Xp11.2 translocation RCC. Based on quantitatively analyzing the distribution of microvessels and lymphatics of Xp11.2 translocation RCC histologically, and comparing it with ccRCC.

## Methods

### Patients

This study was reviewed and approved by the institutional ethics committees of Nanjing Drum Tower Hospital. We reviewed the data of 34 patients diagnosed with Xp11.2 translocation RCC from January 2007 to December 2018, while 77 patients with ccRCC were grouped as the control using the statistic method of propensity score matching (PSM) to keep the homogeneity of the clinicopathological data. The Xp11.2 translocation RCC was diagnosed using both TFE3 immunohistochemistry staining and self-designed break-apart TFE3 fluorescence in situ hybridization probes [14]. All enrolled patients were subjected to radical nephrectomy and did not receive neoadjuvant therapy or adjuvant chemotherapy or radiotherapy. Then, the postoperative pathological staging was assessed using the eighth edition TNM classification issued by the AJCC Staging in 2017. The nuclear grade was determined using the 2016 WHO/ISUP pathological nuclear grading system, whereas clinical staging was based on the findings of preoperative ultrasonography, nuclear medicine bone scanning, computed tomography of the abdomen, and lung radiography. Finally, computed tomography of the lung and brain in conjunction with magnetic resonance imaging of the bone and abdomen were performed as necessary [13]. Integrated survival data and tissue samples were available for all 111 patients enrolled in this study.

### Immunohistochemistry

Formalin-fixed paraffin-embedded tissue sections (thickness 4 μm) were deparaffinized in xylene and rehydrated in graded ethanol series. Next, the deparaffinized slides were placed in boiling saline sodium citrate to repair antigen for 10 min. Then, the specimens were incubated in 3% hydrogen peroxide for 10 min to block endogenous peroxidase activity and also washed with phosphate-buffered saline (PBS) three times. Afterward, all dried sections were incubated in 10% bull serum albumin for 30 min, followed by incubation overnight at 4 °C with primary CD34 monoclonal antibody (Dako) at 1:100 dilution or D2–40 monoclonal antibody (Dako) at 1:100 dilution. The biotinylated secondary antibodies were labeled using the streptomycin avidin-peroxidase solution (Dako) at 1:500 dilution. Lastly, they were stained using Simple DAB Stain Kit (Dako) as per the manufacturer’s instructions, and the sections were counterstained with hematoxylin. The sections from each specimen (processed with nonimmunized mouse serum instead of the primary antibody) were used as negative controls.

### Quantification of D2–40 positive lymph vessels and CD34 positive microvessels

To quantify the MVD, tumor sections stained with CD34 antibody were screened to select the maximum staining area (hot spot) by two experienced pathologists based on the criteria [13] at × 100 magnification (Microscope Product ID 7C86158, Model CX23LEDRFS1C, Olympus Corpn., China). Next, the mean value of CD34 positive microvessels within five “hot spots” for each tumor section was computed at × 400 magnification. Microvessel density (MVD) was defined as the number of microvessels within 0.2 square millimeters (mm^2^). Then, 5 consecutive fields within the “hot spots” at × 400 magnification were captured using a digital image capture device (Microscope digital imaging system, Model DM500, Beijing Jiayuan Industrial Technology Co. LTD., China) for the estimation of MVA. The images resolution is 2592 Pixel Per Inch at width and 1944 Pixel Per Inch at height, and it is unchanged during image processing. Additionally, the MVA was calculated using image analysis software (Image-Pro Plus 6.0, Media Cybernetics, USA) and depicted as the mean lumen area of the microvessels within the five 0.20 mm^2^ fields. The evaluation protocol of LVA and LVD was similar to that of quantifying the microvessels. Finally, intratumoral microvessels and lymphatics were described as those located within the tumor mass, while peritumoral microvessels and lymphatics were outlined as those located within 2 mm outside of the tumor border.

### Statistical analysis

Quantitative data were expressed as mean ± standard deviation and compared using Student′s t-test, whereas qualitative data were presented as counts and percentages, and assessed using either Chi-Square test or Fisher′s exact test. To control the homogeneity of the clinicopathological data between the two groups, the PSM was applied to balance the clinicopathological covariates including laterality, pathological T or N stage, clinical M stage, AJCC stage, WHO/ISUP grade and tumor size. Moreover, we excluded the age and gender of patients between subgroups when performing PSM since they are different naturally [15]. The Kaplan-Meier method of survival analysis was used to estimate cancer-specific survival (CSS), while statistical comparisons were assessed using the log-rank test. The CSS was described as the time interval between the date of surgery and the date of death or last follow-up. Then, the Cox proportional hazards model was used to evaluate the predictive role of the factors that indicated significance in the long-rank test. The significance level was set at *P* < 0.05. All statistical analysis was implemented using SPSS software version 23.0 (IBM SPSS Inc., Chicago, IL, USA). The survival curves were drawn using GraphPad Prism software version 7.0.

## Results

### Clinicopathological data

The clinicopathological features of the two groups (Xp11.2 translocation RCC and ccRCC groups) are shown in Table 1. The mean age was 29.9 ± 13.5 years (ranging from 3 to 64 years) in the Xp11.2 translocation RCC group (male to female ratio of 1:1.62), whereas in the ccRCC group (male to female ratio of 1:0.33) it was 57.7 ± 12.9 years (ranging from 24 to 82 years). Then, the median follow-up time for the Xp11.2 translocation RCC was 63 months (ranging from 15 to 137 months), while the ccRCC group recorded 53 months (ranging from 8 to 94 months). Notably, there was a statistical difference in the age and gender between subgroups (both *P* < 0.001). However, after performing PSM there was no significant difference between the two groups in terms of laterality, pathological T or N stage, clinical M stage, AJCC stage, WHO/ISUP grade and tumor size (all *P* > 0.05).

**Table 1:** Clinicopathological features of patients with both Xp11.2 translocation RCC and ccRCC

Characteristics	Xp11.2 translocation RCC	ccRCC	*P-*value
	No. (%)	No. (%)	
Age			< 0.001
< 45	28 (82.4)	14 (18.2)	
≥ 45	6 (17.6)	63 (81.8)	
Gender			< 0.001
Male	13 (38.2)	58 (75.3)	
Female	21 (61.8)	19 (24.7)	
Laterality			0.093
Right	20 (58.8)	32 (41.6)	
Left	14 (41.2)	45 (58.4)	
Pathological T stage			0.654
pT1/pT2	29 (85.3)	63 (81.8)	
pT3/pT4	5 (14.7)	14 (18.2)	
Pathological N stage			0.251
pN_0_	27 (79.4)	69 (89.6)	
pN_1_	7 (20.6)	8 (10.4)	
Clinical M stage			0.999
cM_0_	32 (94.1)	71 (92.2)	
cM_1_	2 (5.9)	6 (7.8)	
AJCC stage			0.982
I/II	27 (79.4)	61 (79.2)	
III/IV	7 (20.6)	16 (20.8)	
WHO/ISUP grade			0.401
1/2	24 (70.6)	48 (62.3)	
3/4	10 (29.4)	29 (37.7)	
Tumor size (cm)	5.5 ± 2.7	5.6 ± 2.3	0.806

*Xp11.2 translocation RCC* Xp11.2 translocation renal cell carcinoma, *ccRCC* Clear cell renal cell carcinoma, *AJCC* American Joint Committee On Cancer, *WHO/ISUP* World Health Organization/International Society of Urological Pathology

### Distribution of microvessels and lymphatic vessels in Xp11.2 translocation RCC

**Fig. 1:**
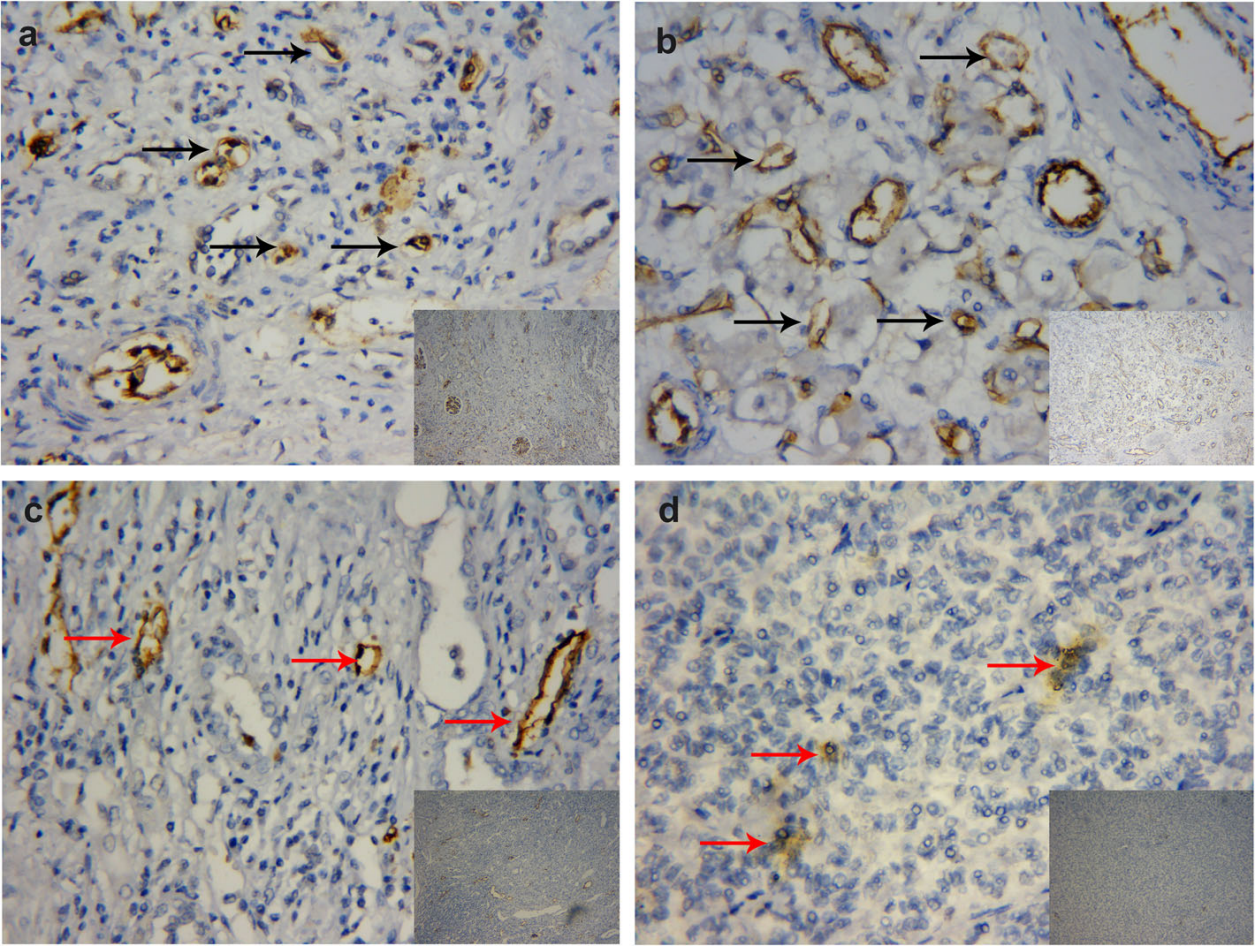
Representative immunostaining for CD34-positive microvessels (**a**, **b**, black arrowheads) and D2–40-positive lymphatic vessels (**c**, **d**, red arrowheads) at × 100 and × 400 magnification in Xp11.2 translocation renal cell carcinoma. **a** Peritumoral microvessels, **b** Intratumoral microvessels, **c** Peritumoral lymphatic vessels, and **d** Intratumoral lymphatic vessels

**Fig. 2:**
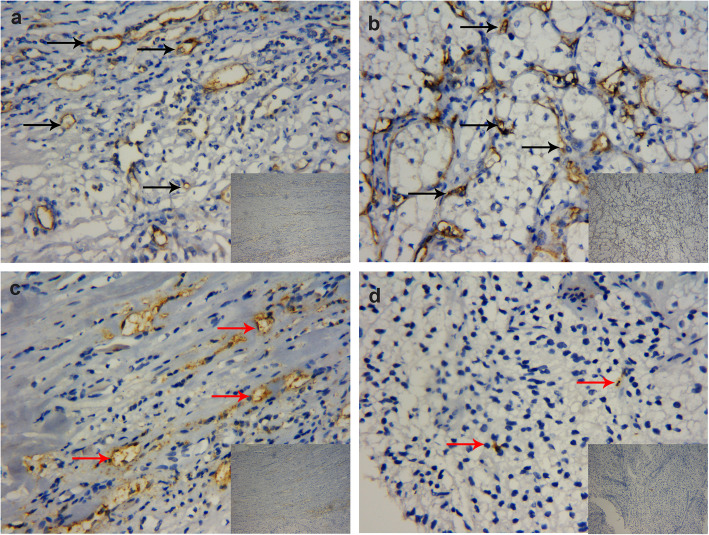
Representative immunostaining for CD34-positive microvessels (**a**, **b**, black arrowheads) and D2–40-positive lymphatic vessels (**c**, **d**, red arrowheads) at × 100 and × 400 magnification in clear cell renal cell carcinoma. **a** Peritumoral microvessels, **b** Intratumoral microvessels, **c** Peritumoral lymphatic vessels, and **d** Intratumoral lymphatic vessels

The CD34-positive microvessels were examined microscopically throughout the intratumoral and peritumoral area of Xp11.2 translocation RCC and ccRCC (Fig. 1a, b and 2a, b). Plenty of CD34-positive microvessels in the intratumoral and peritumoral area were counted (Table 2), but the MVD and MVA of Xp11.2 translocation RCC in the two detected areas (intratumoral and peritumoral area) were not significantly different from those of ccRCC (all *P* > 0.05). Furthermore, in particular, the MVD and MVA of the intratumoral area in Xp11.2 translocation RCC were significantly different compared to those of the peritumoral area (both *P* < 0.05). On the other hand, D2–40-positive lymphatic vessels were assessed microscopically throughout the two detected areas of Xp11.2 translocation RCC and ccRCC (Fig. 1c, d and 2c, d). Afterward, D2–40-positive lymphatic vessels in the peritumoral area were detected in all Xp11.2 translocation RCC specimens, while the same vessels in the intratumoral area were recorded in only 9 (26.5%) Xp11.2 translocation RCC cases. The values presented in Table 2 also demonstrated that the peritumoral LVD and LVA of Xp11.2 translocation RCC were higher than those of ccRCC (all *P* < 0.05). Nonetheless, the intratumoral LVD and LVA of Xp11.2 translocation RCC were not statistically different compared to that of ccRCC (both *P* > 0.05). Lastly, the peritumoral LVD and LVA in Xp11.2 translocation RCC were significantly higher than those of the intratumoral area (both *P* < 0.05) (Table 2).

**Table 2:** Distribution characteristics of microvessels and lymphatic vessels in both Xp11.2 translocation RCC and ccRCC

Histological subtypes		Xp11.2 translocation RCC	ccRCC	*P-*value
		Mean ± SD	Mean ± SD	
Intra-tumor	MVD	157 ± 10^*^	153 ± 12^*^	0.723
	MVA	44,259 ± 8701^*^	45,665 ± 10559^*^	0.649
	LVD	1 ± 2^*^	2 ± 2^*^	0.692
	LVA	535 ± 563^*^	481 ± 465^*^	0.680
Peri-tumor	MVD	108 ± 10	110 ± 11	0.247
	MVA	34,975 ± 6131	37,009 ± 6841	0.362
	LVD	10 ± 3	8 ± 3	0.040
	LVA	3326 ± 1568	2556 ± 1120	0.029

*Xp11.2 translocation RCC* Xp11.2 translocation renal cell carcinoma, *ccRCC* Clear cell renal cell carcinoma, *MVD* Microvascular density, *MVA* Microvascular area, *LVA* Lymph vessel area, *LVD* Lymph vessel density, *SD* Standard deviation; ^*^showed the difference of parameters between intra-tumor and peri-tumor, *P* < 0.05

### Association between pathological variables and MVD, MVA, LVD or LVA

As shown in Table 3, the MVD and MVA (but not the LVD or LVA) of the intratumoral area was significantly associated with pT stage (*P* = 0.023 and *P* < 0.001, respectively), pN stage (both *P* < 0.001), cM stage (*P* = 0.017 and *P* = 0.001, respectively), AJCC stage (both *P* < 0.001), and WHO/ISUP grade (*P* = 0.008 and *P* = 0.002, respectively). Although we also analyzed the correlation of pathological variables with MVD or MVA in the peritumoral area, no correlation was noted (all *P* > 0.05) (Table 4). Conversely, the values presented in Table 4 revealed that the peritumoral LVD or LVA was significantly associated with several pathological variables namely pT stage (*P* = 0.004 and *P* = 0.001, respectively), pN stage (*P* = 0.001 and *P* = 0.011, respectively), cM stage (*P* = 0.009 and *P* = 0.002, respectively), AJCC stage (*P* = 0.001 and *P* = 0.011, respectively), and WHO/ISUP grade (*P* = 0.007 and *P* = 0.002, respectively).

**Table 3:** Relationship between MVD, MVA, LVD or LVA and pathological variables in intratumoral area of Xp11.2 translocation RCC

Variables	No.	Intra-tumor (Mean ± SD)							
		MVD	*P*-value	MVA	*P-*value	LVD	*P-*value	LVA	*P-*value
Pathological T stage			0.023		< 0.001		0.718		0.664
pT1/pT2	29	156 ± 10		41,933 ± 6967		1 ± 2		553 ± 602	
pT3/pT4	5	166 ± 4		57,750 ± 4244		1 ± 1		431 ± 260	
Pathological N stage			< 0.001		< 0.001		0.615		0.911
pN_0_	27	154 ± 9		41,212 ± 6658		1 ± 2		529 ± 604	
pN_1_	7	168 ± 5		56,008 ± 4630		2 ± 2		557 ± 408	
Clinical M stage			0.017		0.001		0.690		0.837
cM_0_	32	156 ± 10		43,329 ± 8083		1 ± 2		540 ± 575	
cM_1_	2	168 ± 1		59,139 ± 1477		1 ± 1		454 ± 445	
AJCC stage			< 0.001		< 0.001		0.615		0.911
I/II	27	154 ± 9		41,212 ± 6658		1 ± 2		529 ± 604	
III/IV	7	168 ± 5		56,008 ± 4630		2 ± 2		557 ± 408	
WHO/ISUP grade			0.008		0.002		0.891		0.710
1/2	24	154 ± 9		41,463 ± 7031		1 ± 2		559 ± 635	
3/4	10	164 ± 9		50,969 ± 8965		2 ± 1		478 ± 358	

*MVD* Microvascular density, *MVA* Microvascular area, *LVA* Lymph vessel area, *LVD* Lymph vessel density, *AJCC* American Joint Committee On Cancer, *WHO/ISUP* World Health Organization/International Society of Urological Pathology, *Xp11.2 translocation RCC* Xp11.2 translocation renal cell carcinoma

**Table 4:** Relationship between MVD, MVA, LVD or LVA and clinicopathological variables in peritumoral area of Xp11.2 translocation RCC

Variable	No.	Peri-tumor (Mean ± SD)							
		MVD	*P-*value	MVA	*P-*value	LVD	*P-*value	LVA	*P-*value
Pathological T stage			0.399		0.563		0.004		< 0.001
pT1/pT2	29	107 ± 10		34,718 ± 6183		9 ± 3		2914 ± 1125	
pT3/pT4	5	111 ± 8		36,468 ± 6259		13 ± 3		5719 ± 1746	
Pathological N stage			0.397		0.569		0.001		0.011
pN_0_	27	106 ± 10		34,201 ± 6083		9 ± 3		2837 ± 1089	
pN_1_	7	114 ± 8		37,960 ± 5767		13 ± 3		5212 ± 1778	
Clinical M stage			0.419		0.175		0.009		0.002
cM_0_	32	108 ± 10		34,713 ± 6223		9 ± 3		3125 ± 1381	
cM_1_	2	114 ± 4		39,169 ± 1592		13 ± 2		6539 ± 356	
AJCC stage			0.059		0.151		0.001		0.011
I/II	27	106 ± 10		34,201 ± 6083		9 ± 3		2837 ± 1089	
III/IV	7	114 ± 8		37,960 ± 5767		13 ± 3		5212 ± 1778	
WHO/ISUP grade			0.103		0.181		0.007		0.002
1/2	24	106 ± 10		34,059 ± 6357		9 ± 3		2822 ± 1146	
3/4	10	112 ± 9		37,174 ± 5189		12 ± 3		4536 ± 1833	

*MVD* Microvascular density, *MVA* Microvascular area, *LVA* Lymph vessel area, *LVD* Lymph vessel density, *AJCC* American Joint Committee On Cancer, *WHO/ISUP* World Health Organization/International Society of Urological Pathology, *Xp11.2 translocation RCC* Xp11.2 translocation renal cell carcinoma

### Correlation between clinical prognosis and MVD, MVA, LVD, and LVA

**Fig. 3:**
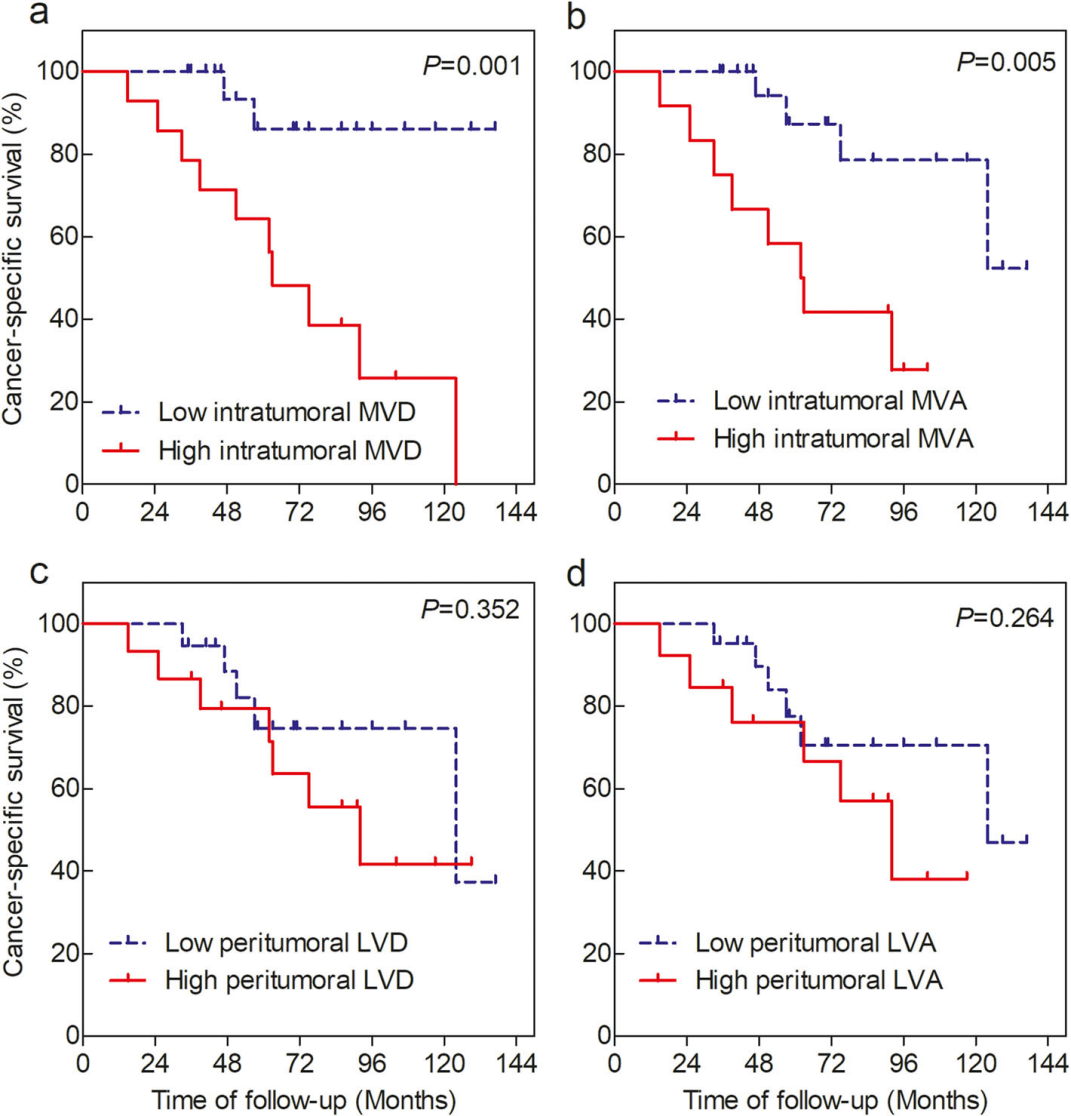
Kaplan-Meier method plotting cancer-specific survival (CSS) curves in Xp11.2 translocation renal cell carcinoma patients grouped by intratumoral MVD, intratumoral MVA, peritumoral LVD, and peritumoral LVA, stratified according to the median cut-off values. **a** CSS for patients with low and high intratumoral MVD, **b** CSS for patients with low and high intratumoral MVA, **c** CSS for patients with low and high peritumoral LVD, and **d** CSS for patients with low and high peritumoral LVA

The threshold level of MVD, MVA, LVD, or LVA was characterized according to the median. The median cut-off values for intratumoral MVD and MVA were 157 and 44,259, while for peritumoral LVD and LVA were 10 and 3326, respectively. High and low group values were defined based on the median cut-off value. Kaplan–Meier analysis of CSS in patients with intratumoral MVD or MVA (Fig. 3a and b) revealed that CSS was significantly longer in patients with low intratumoral MVD or MVA than in patients with high intratumoral MVD or MVA (*P* = 0.001 and *P* = 0.005, respectively). On the other hand, peritumoral LVD or LVA was not associated with CSS in patients with both low peritumoral LVD or LVA and high peritumoral LVD or LVA in the Fig. 3c and d (*P* = 0.352 and *P* = 0.264, respectively). Lastly, multivariate analysis using the Cox proportional hazards model for CSS in Table 5 elucidated that both intratumoral MVD or MVA, and peritumoral LVD or LVA were not independent prognostic factors (all *P* > 0.05).

**Table 5:** Univariate and multivariate analysis of variables predicting CSS

Variables	CSS					
	Univariate analysis			Multivariate analysis		
	HR	95%CI	*P-*value	HR	95%CI	*P-*value
AJCC stage (I/II vs III/IV)	5.595	1.697–18.449	0.005	5.381	1.157–16.858	0.039
WHO/ISUP grade (1/2 vs 3/4)	3.303	1.039–10.505	0.043	0.292	0.017–5.073	0.398
Intratumoral MVD (< 157 vs ≥157)	7.989	1.748–36.502	0.007	6.906	0.948–50.329	0.057
Intratumoral MVA (< 44,259 vs ≥44,259)	5.378	1.424–20.306	0.013	1.428	0.196–10.390	0.725
Peritumoral LVD (< 10 vs ≥10)	1.721	0.541–5.470	0.358	0.572	0.046–7.043	0.572
Peritumoral LVA (< 3326 vs ≥3326)	1.950	0.591–6.433	0.273	1.230	0.136–11.125	0.854

*HR* Hazard ratio, *CI* Confidence interval, *MVD* Microvascular density, *MVA* Microvascular area, *CSS* Cancer-specific survival, *LVA* Lymph vessel area, *LVD* Lymph vessel density, *AJCC* American Joint Committee On Cancer, *WHO/ISUP* World Health Organization/International Society of Urological Pathology

## Discussion

Angiogenesis and lymphangiogenesis within malignant tumors are crucial for tumor growth and metastasis [1, 2]. Numerous studies have confirmed that tumor micro-angiogenesis and lymphangiogenesis could be effective prognostic predictors in many malignant tumors such as prostatic adenocarcinoma, thyroid cancer, and breast cancer [16–18]. On the contrary, other studies based on meta-analysis showed that tumor micro-angiogenesis and lymphangiogenesis are not useful predictive factors for the prognosis in RCC [19, 20]. However, Xp11.2 translocation RCC is a rare subtype of RCC, and its clinical outcome is significantly different from the common subtypes of RCC. The Xp11.2 translocation RCC has been noted to depict a wide variation in biological behavior and clinical outcome [7]. Therefore, the role of micro-angiogenesis and lymphangiogenesis within Xp11.2 translocation RCC is imperative, and necessities further studies. Although this study has demonstrated that tumor-related microvascular and lymphatic parameters were not independent prognostic factors for Xp11.2 translocation RCC patients, we revealed that Xp11.2 translocation RCC is a highly vascularized solid RCC, which is characterized by rich lymph vessels in the peritumoral area. This valuable information may provide a histological theoretical basis for tumor metastatic pathways and thereby giving new strategies for treating Xp11.2 translocation RCC based on the tumor anti-angiogenic therapy theory.

The tumor micro-angiogenesis has been measured in several ways including MVD, MVA, quantifying tumor angiogenic molecules, microvessel invasion and assessing the presence of tumor-associated angiogenic receptors, while the lymphangiogenesis has been quantified using various methods such as LVD, LVA, lymphatic vessel invasion, and vascular endothelial growth factors family members [10, 12, 21–23]. Currently, the MVD, MVA, LVD, and LVA have widely been used as measurement parameters for assessing micro-angiogenesis and lymphangiogenesis within RCC. In this context, we computed the microvessels and lymphatics within the Xp11.2 translocation RCC using MVD, MVA, LVD, and LVA. Previous studies have opined that there were many vascular markers such as factor VIII, CD31, CD34, and CD105 for marking microvessels endothelium in malignant tumors [12]. Overall, findings from multiple studies have noted that CD34 expression exhibited no variation over time and thus better compared to several other markers for staining microvessels [24, 25]. Herein, CD34 was used as the most suitable vascular markers for counting microvessels in Xp11.2 translocation RCC. Furthermore, other several lymphatic vessel markers namely D2–40, lymphatic vessel endothelial hyaluronan receptors, and vascular endothelial growth factor receptors are available for detecting lymphatic endothelium [10, 17]. In particular, the D2–40 antibody was used to mark lymphatic vessels in this study due to its specificity for lymphatic vessels [26].

Here, the prognostic value of MVD and MVA indicating the microvessels in RCC remained inconsistent and unclear. In another study by Sharma et al. reported that the MVD within RCC was not significantly correlated with tumor grade and stage, and thereby MVA appeared to be a good prognostic factor for RCC [12]. Elsewhere, multiple studies demonstrated that MVD in RCC exhibited an inverse association with tumor aggressiveness [24, 27], whereas other reports revealed no relationship between MVD and survival [19, 20]. In this work, the intratumoral MVD and MVA were significantly associated with grade and stage of Xp11.2 translocation RCC. However, the intratumoral MVD and MVA were not independent prognostic factors for Xp11.2 translocation RCC. The discrepancy between the results of these studies could be attributed to various factors such as tumor vascular system complexity, pathological subtypes of RCC, sample size, selection of vascular markers, immunohistochemistry staining quality, and methods of microvessel count. Despite different kinds of research focused on the tumor micro-angiogenesis in RCC, the prognostic value of LVD and LVA for assessing the lymphangiogenesis in RCC are rarely studied. Moreover, Iwata et al. reported that both intratumoral and peritumoral LVD were not associated with the pathological features in RCC [10]. On the other hand, a recent study has elucidated that the presence of intratumoral lymphatic vessels exhibited a significant correlation with distant metastasis and lymph node metastasis [28]. In contrast, findings from our study demonstrated that peritumoral LVD or LVA of Xp11.2 translocation RCC was significantly associated with several pathological variables including pT stage, pN stage, cM stage, AJCC stage, and WHO/ISUP grade, while increased peritumoral LVD or LVA indicated a relationship with high pathological stage and increasing nuclear grade. Notably, pathological subtypes of RCC, sample size, immunohistochemistry staining quality, and methods of microvessel count may be major reasons why conclusions from the above studies were very different. Therefore, the role of tumor lymphangiogenesis in the intratumoral and peritumoral area of RCC necessitates further research.

To avoid the selection bias of different areas of the tumor examined under the microscope, we analyzed the intratumoral and peritumoral areas of Xp11.2 translocation RCC. The results revealed that MVD or MVA in the intratumoral area was significantly higher compared to that of the peritumoral area, which was inconsistent with the findings of Cao et al. [29]. In particular, their study implied that the peritumoral area in ccRCC included considerably more microvessels. The inconsistency between these two studies is likely due to pathological subtypes of RCC and microvessel counting methods. Additionally, CSS in Xp11.2 translocation RCC patients were significantly longer in patients with low intratumoral MVD or MVA than in patients with high intratumoral MVD or MVA, which corresponded with the result of a previous study [11]. Nevertheless, another recent study has revealed that high MVD was associated with longer survival, which contradicts the findings of our study [13]. Conclusively, MVD or MVA in the intratumoral area plays a minimal role in predicting the prognosis for Xp11.2 translocation RCC, but it is not an independent prognostic factor for Xp11.2 translocation RCC. Compared with the tumor microvessels in intratumoral and peritumoral areas of Xp11.2 translocation RCC, the tumor lymphangiogenesis was significantly different between intratumoral area and peritumoral area of Xp11.2 translocation RCC. Besides, findings from this work indicated a predominance of D2–40 positive lymph vessels primarily in the peritumoral area of Xp11.2 translocation RCC, whereas only 9 (26.5%) cases depicted D2–40 positive lymph vessels in the intratumoral area of Xp11.2 translocation RCC. Recently, many studies have recorded intratumoral and peritumoral distribution of lymphatic vessels in RCC [10, 28]. Interestingly, the distribution of lymphatic vessels in Xp11.2 translocation RCC was consistent with that of traditional RCC. High-tissue pressure in tumor interstitium could be the main reason why lymph vessels were not detected in the intratumoral area of RCC [17]. Lastly, this study outlined that peritumoral LVD or LVA was not associated with CSS in patients with both low peritumoral LVD or LVA and high peritumoral LVD or LVA. However, previous reports indicated that RCC patients with low peritumoral LVD exhibited significantly shorter CSS [30]. Surprisingly, the role of tumor lymphangiogenesis in RCC remains elusive, and hence further studies are indispensable.

There are some limitations in the study. The sample size was not enough large due to the low incidence of this rare disease, and the follow-up time for patients was relatively short. In addition, the criteria for defining microvessels need to be further clarified, whereas the counting method should be more objective, accurate, convenient and efficient.

## Conclusions

Overall, findings from this study indicate that Xp11.2 translocation RCC is a highly vascularized solid RCC, characterized by rich lymph vessels in the peritumoral area. Inhibiting tumor lymphangiogenesis and micro-angiogenesis would delay tumor growth and metastasis, which may provide novel strategies for treating Xp11.2 translocation RCC. Although MVD, MVA, LVD, or LVA cannot be used as novel risk factors predicting prognosis for Xp11.2 translocation RCC patients, high intratumoral MVA and MVD indicated poor prognosis and thereby they may be important to clinicians.

## Data Availability

The datasets analyzed during the current study are available from the corresponding author on reasonable request.

## Abbreviations

RCC: Renal cell carcinoma
Xp11.2 translocation RCC: Xp11.2 translocation renal cell carcinoma
ccRCC: Clear cell renal cell carcinoma
MVD: Microvessel density
MVA: Microvessel area
LVD: Lymph vessel density
LVA: Lymph vessel density
CSS: Cancer-specific survival
PSM: Propensity score matching
